# Comparison of three frailty scales for prediction of prolonged postoperative ileus following major abdominal surgery in elderly patients: a prospective cohort study

**DOI:** 10.1186/s12893-024-02391-6

**Published:** 2024-04-16

**Authors:** Xianwei Xiong, Ting Zhang, Huan Chen, Yiling Jiang, Shuangyu He, Kun Qian, Hui Li, Xiong Guo, Juying Jin

**Affiliations:** 1 Department of Anesthesiology, The First Affiliated Hospital of Chongqing Medical University, 1 Youyi Road, Chongqing, 400016 China; 2 Department of Gastrointestinal Surgery, The First Affiliated Hospital of Chongqing Medical University, 1 Youyi Road, Chongqing, 400016 China

**Keywords:** Prolonged postoperative ileus, Frailty, Elderly patients, Prediction, Major abdominal surgery

## Abstract

**Background:**

To determine whether frailty can predict prolonged postoperative ileus (PPOI) in older abdominal surgical patients; and to compare predictive ability of the FRAIL scale, the five-point modified frailty index (mFI-5) and Groningen Frailty Indicator (GFI) for PPOI.

**Methods:**

Patients (aged ≥ 65 years) undergoing major abdominal surgery at our institution between April 2022 to January 2023 were prospectively enrolled. Frailty was evaluated with FRAIL, mFI-5 and GFI before operation. Data on demographics, comorbidities, perioperative management, postoperative recovery of bowel function and PPOI occurrence were collected.

**Results:**

The incidence of frailty assessed with FRAIL, mFI-5 and GFI was 18.2%, 38.4% and 32.5% in a total of 203 patients, respectively. Ninety-five (46.8%) patients experienced PPOI. Time to first soft diet intake was longer in patients with frailty assessed by the three scales than that in patients without frailty. Frailty diagnosed by mFI-5 [Odds ratio (OR) 3.230, 95% confidence interval (CI) 1.572–6.638, *P* = 0.001] or GFI (OR 2.627, 95% CI 1.307–5.281, *P* = 0.007) was related to a higher risk of PPOI. Both mFI-5 [Area under curve (AUC) 0.653, 95% CI 0.577–0.730] and GFI (OR 2.627, 95% CI 1.307–5.281, *P* = 0.007) had insufficient accuracy for the prediction of PPOI in patients undergoing major abdominal surgery.

**Conclusions:**

Elderly patients diagnosed as frail on the mFI-5 or GFI are at an increased risk of PPOI after major abdominal surgery. However, neither mFI-5 nor GFI can accurately identify individuals who will develop PPOI.

**Trial registration:**

This study was registered in Chinese Clinical Trial Registry (No. ChiCTR2200058178). The date of first registration, 31/03/2022, https://www.chictr.org.cn/.

## Background

Postoperative ileus (POI) is defined as temporary reduction in gastrointestinal motility following surgery. POI is featured by inability to oral intake, nausea and vomiting, persistent abdominal distension and pain, and delayed passage of flatus and stool [1, 2]. Although POI may last for longer duration or reoccur, it usually resolves within 2–4 days. When the symptoms persist longer than expected duration, it is considered as prolonged postoperative ileus (PPOI) [3]. According to the results of the previous literature, PPOI is proposed to be defined as ileus that develops 4 postoperative days, which has been adopted by many researchers [4]. As a common complication after abdominal surgery, the incidence of PPOI is reported to be between 10 and 40% [5–7]. PPOI has been shown to be associated with delayed recovery after surgery, longer length of stay, decrease in quality of life, and higher health care expenses [8, 9]. As options for the treatment of PPOI are limited, efforts to predict it and to reduce its duration should start before operation.

Frailty is described as a state of being vulnerable to stress resulted from age-related functional declines in multi-systems [10]. Frailty has been shown to be strongly and consistently associated with adverse postoperative outcomes in the setting of major procedures, such as occurrence of major adverse clinical events, length of stay, and 30-day mortality [11–14]. However, most surgical studies investigating frailty focus on hospitalization variables, there are few studies addressing the association of frailty with PPOI in elderly patients undergoing abdominal surgery.

An ideal frailty assessment scales should be able to identify frailty and to predict poor outcomes accurately [15]. Although more than 70 frailty indices have been developed, optimal measures to frailty evaluation have not been identified [16]. Among all of the frailty scales, FRAIL scale is highly cited in the research literature; the five-point modified frailty index (mFI-5) is one of the commonly used scales [17–19]; and the Groningen Frailty Indicator (GFI) is a multi-dimensional including disability and morbidity [20, 21]. However, research comparing FRAIL, mFI-5 and GFI among elderly patients undergoing abdominal surgery for prediction of bowel function recovery are limited. We therefore performed this prospective cohort study to establish whether preoperative frailty is able to predict PPOI in older patients undergoing major abdominal surgery; and to compare predictive ability of the three frequently used frailty scales for PPOI in this population.

## Methods

### Study design and participants

We carried out a prospective cohort study in patients (aged ≥ 65 years) who scheduled to receive elective radical resection of gastroenteric tumors under general anesthesia from April 2022 to January 2023. Exclusion criteria were patients who declined to participate in the study; patients with constipation, irritable bowel syndrome, and other diseases affecting bowel function; patients who were unable to communicate because of dementia, mental disorders, or language barrier; American Society of Anesthesiologists (ASA) physical status classification > IV; patients scheduled for open procedures; Patients receiving reoperation within postoperative 4 days were excluded from data analysis.

The present study was approved by the Ethics Committee of our institution (No. 2021 − 700), and registered in Chinese Clinical Trial Registry (No. ChiCTR2200058178, 31/03/2022). All subjects provided written informed consent.

### Baseline information

Baseline evaluation was conducted at the first interview by a trained investigator, which included demographic data on age, sex, and body mass index (BMI), status of smoking, alcohol drinking, previous abdominal surgery, comorbidities like hypertension, diabetes, coronary disease, chronic obstructive pulmonary disease (COPD) and cerebrovascular disease, ASA physical status classification, smoking status, and frailty status based on the three frailty instruments (FRAIL, mFI-5 and GFI).

The FRAIL scale was conceptualized by the International Association of Nutrition and Aging task force in 2008 [22]. It contains 5 items: fatigue, resistance, illnesses, ambulation and weight loss. The FRAIL scale gives 1 score ranges from 0 to 5, 1 point for each item. Patients are considered as frail at a score ≥ 3 points. The FRAIL has been fully validated in diverse populations including community-dwelling and hospitalized older adults in Australia, United States, and China [23–26].

The mFI-5 was calculated according to the 5 items as developed in 2018: hypertension, diabetes, COPD, congestive heart failure, and dependent physical status [27]. Presence of each item is counted by a 1-point value, which leads to the scores range from 0 to 5. A cut-point of ≥ 2 indicates frailty. This instrument has been cross-culturally adapted and fully validated in Chinese population [28, 29].

The self-report GFI evaluates the multidimensional feature of frailty based on a conceptual model including physical, psychological and social domains [20]. The scale consists of 15 items, with scores ranged between 0 and 15. A cut-point of ≥ 4 is considered as an indicator of frailty. The psychometric properties of GFI have been sufficiently validated in both community-dwelling and hospitalized elderly individuals in China, Germany, and the Netherlands [30–33].

### Surgical and anesthetic management

Anesthetic and surgical techniques were carried out according to the protocols routinely used in our institution. No premedication was given. Standard monitoring during operation including electrocardiogram, pulse oxygen saturation, and noninvasive blood pressure was established for each patient. Central venous pressure and invasive arterial pressure were monitored when needed.

General anesthesia was induced with sufentanil, propofol, and neuromuscular blockers (rocuronium or vecuronium). Infusion of propofol and inhalation of sevoflurane were employed for anesthesia maintenance. Neuromuscular blockers were injected when necessary and were stopped for ≥ 30 min prior to the closure of the incision. Bispectral index (BIS) was monitored during maintenance of general anesthesia. The depth of anesthesia was targeted to maintain BIS at the level of 40–55.

A serotonin receptor antagonist was given at the end of the surgery. Patients were delivered to the post-anesthesia care unit (PACU) after being extubated the in the operating room. Nasogastric tube was routinely discontinued in the operating room. Intraoperative variables including surgical type, operation time, fluid and packed red blood cell (RBC) administration, volume of blood loss and urine output were collected.

After arrival to PACU, postoperative pain relief was provided by intravenous sufentanil patient-controlled analgesia(PCA)via an infusion pump (Rythmic™ Evolution, Micrel, Athens, Greece ) (bolus 2 ug, lockout 10 min, no basal infusion). Patients were cared in PACU for ≥ 30 min and subsequently transferred to the ward if the Steward score was ≥ 4.

Postoperative management strategies were generally based on ERAS protocol and partly adapted according to patients’ conditions and their attending surgeons. Dietary supplementation products containing dietary fiber, glutamine, and oligosaccharide were provided on postoperative day (POD) 1, and then a soft diet was added on POD 2–3. Patients were given abdominal massage if the time to first flatus was ≥ 48 h. Parenteral nutrition was administered when the time to first flatus was ≥ 72 h. Patients were instructed by physical therapists to mobilize and walk around the ward on POD 1. Abdominal drainage tubes and urinary catheter were discontinued as soon as possible.

### Outcome measures

The primary outcome was the occurrence of PPOI. It was defined based on previous literature [4, 34]. PPOI was diagnosed when patients had ≥ 2 of the following 5 criteria on POD 4 or more postoperatively: (1) Nausea/vomiting over the preceding 12 h; (2) Intolerance to a solid/semi-solid diet over the preceding 24 h; (3) Persistent abdominal distension; (4) Absence of passage of both flatus and stool over the preceding 24 h; (5) Ileus confirmed on abdominal plain films or CT scans. Secondary outcomes were postoperative recovery of bowel function including time to first flatus, time to first defecation, time to first soft diet intake; and length of hospital stay. Outcome assessors were blinded to frailty status.

### Statistical analysis

Normality of distribution of the continuous data was tested by the Kolmogorov–Smirnov method. Normal distribution data were shown as mean ± standard deviation (SD), and differences between groups were compared by independents sample t-test. Non-normal distribution data were shown as median (range), and differences between groups were compared by the Mann–Whitney U test. Categorical data were shown as number (percentage), and differences between groups were compared by chi-square test. The Cohen’s κ coefficient was calculated to examine the agreement between the frailty scales. The association between preoperative frail status and PPOI was initially analyzed using univariate analysis. Variables were taken into multivariate logistic regression when *P* < 0.20 in univariate analysis to determine the independent risk factors for PPOI.

Receiver operating characteristic (ROC) curves were adopted to determine the predictive ability of each frailty scale against PPOI. Area under curve (AUC) > 0.70 was considered as an indicator that the scale had a good discriminatory value [35]. All analyses were two-tailed, with an α level of 0.05 to determine significance. Data analyses were carried out using SPSS 26 Inc. (Chicago, IL, USA).

## Results

**Fig. 1:**
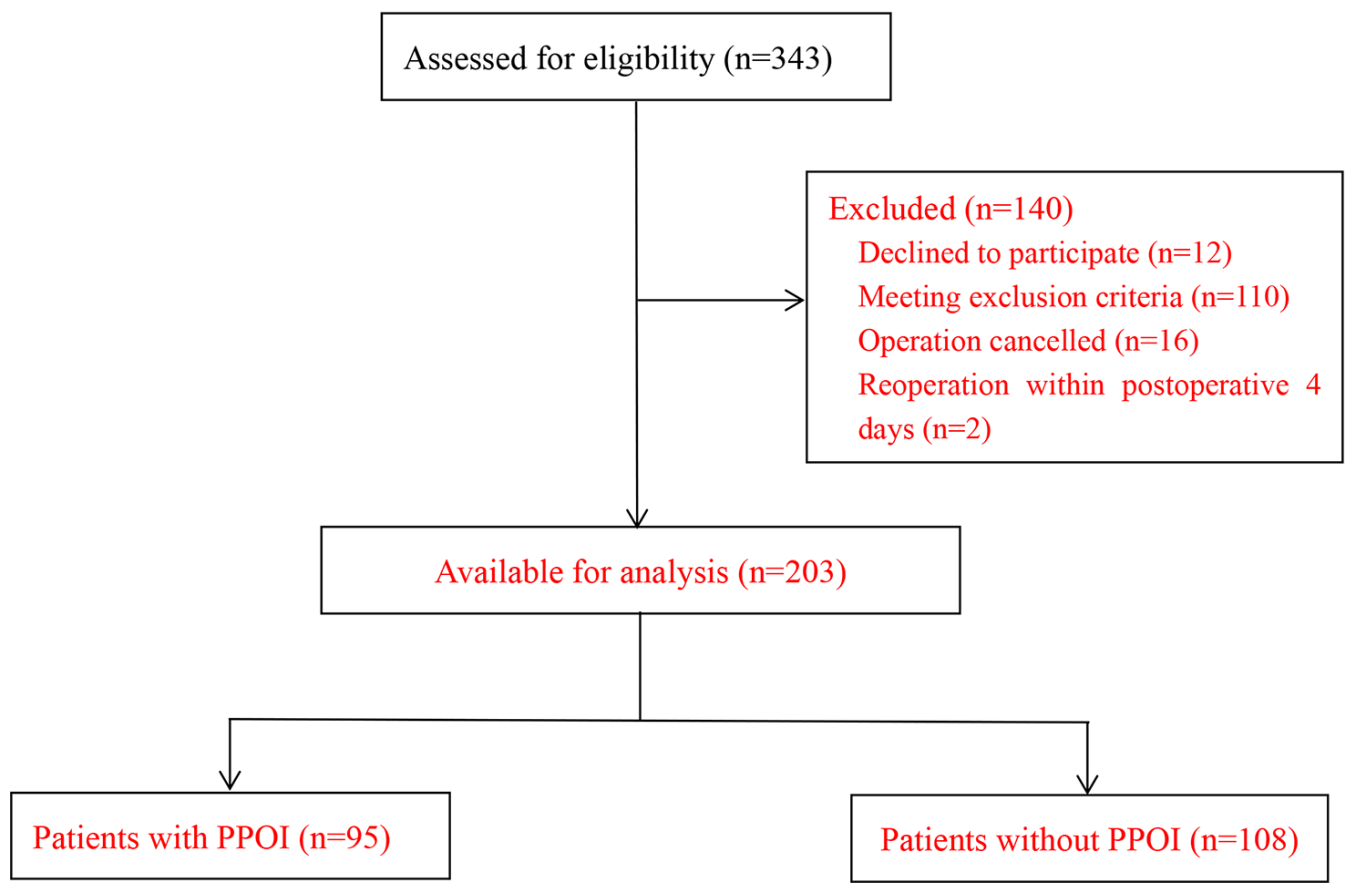
Flowchart of participants throughout the study

Three hundred forty-three patients agreed to be evaluated for inclusion. Of these, 12 patients declined participation, 110 were excluded as per exclusion criteria, 16 surgeries were canceled, and 2 patients were re-operated within 4 days after surgery, leaving a total of 203 were included for analysis (Fig. 1).

### Baseline characteristics

Patient demographics and baseline characteristics are shown in Table 1. Mean age of the subjects was 72.6 years, and 54.7% of them were male. The most commonly included surgeries were colonic procedures. The prevalence of frailty was 18.2% according to the FRAIL scale. Compared to the FRAIL, higher proportions of patients were categorized as frail by mFI-5 scale and GFI index (38.4% for mFI-5 and 32.5% for GFI, respectively).

**Table 1:** Demographic characteristics, surgical and analgesia variables (*n* = 203)

Variables	Value
Age (years, mean ± SD)	72.6 ± 5.8
Male (n, %)	111 (54.7)
BMI (Kg/m^2^, mean ± SD)	22.6 ± 3.2
Smoking (n, %)	70 (34.5)
Alcohol consumption (n, %)	100 (49.3)
Previous abdominal surgery (n, %)	37 (18.2)
Hypertension (n, %)	90 (44.3)
Diabetes (n, %)	31 (15.3)
Coronary heart disease (n, %)	17 (8.4)
COPD (n, %)	73 (36.0)
Cerebral vascular disease (n, %)	15 (7.4)
ASA physical status (n, %)	
I	32 (15.8)
II	136 (67.0)
III	32 (15.8)
IV	3 (1.5)
FRAIL ≥ 3 (n, %)	37 (18.2)
mFI-5 ≥ 2 (n, %)	78 (38.4)
GFI ≥ 4 (n, %)	66 (32.5)
Surgical type (n, %)	
Gastric	41 (20.2)
Small bowel	6 (3.0)
Colonic	110 (54.2)
Rectal	46 (22.7)
Duration of surgery (min, mean ± SD)	209 ± 81
Intraoperative fluid infusion (mL/Kg·h, mean ± SD)	7.3 ± 2.5
Intraoperative packed RBC infusion [mL, median (range)]	0 (0, 800)
Urine output (mL/Kg·h, mean ± SD)	1.9 ± 2.1
Blood loss [mL, median (range)]	50 (5, 600)
Time to first flatus (h, mean ± SD)	55 ± 32
Time to first defecation (h, mean ± SD)	88 ± 42
Time to first soft diet intake (h, mean ± SD)	70 ± 47
PPOI (n, %)	95 (46.8)
Length of hospital stay (d, mean ± SD)	14 ± 6

*ASA* American Society of Anesthesiologists, *BMI* body mass index, *COPD* chronic obstructive pulmonary disease, *FFP* fresh frozen plasma, *GFI* Groningen Frailty Indicator, *mFI-5* five-point modified frailty index, *PPOI* prolonged postoperative ileus, *RBC* red blood cell, *SD* standard deviations

### The agreement among the three frailty scales

Of the 203 subjects, 32 patients (15.8%) were assessed as frail by one scale, 31 (15.3%) by two scales, and 29 (14.3%) by all of the three scales. The Cohen’s κ coefficient was highest between mFI-5 and GFI (mFI-5 and GFI: 0.614, *P* < 0.001; mFI-5 and FRAIL: 0.411, *P* < 0.001; GFI and FRAIL: 0.506, *P* < 0.001).

### Postoperative bowel function recovery and incidence of PPOI

Postoperative recovery of bowel function by frailty status based on each scale is shown in Table 2. Among 203 patients, 95 (46.8%) patients experienced PPOI. The time to first soft diet intake was longer in frail patients than those in non-frail patients assessed by the three scales. The PPOI occurrence was higher in frail patients as defined by the mFI-5 and GFI. Moreover, frail older individuals identified according to the three instruments had significantly increased length of hospital stay when compared to non-frail peers.

**Table 2:** Recovery of bowel function after surgery, PPOI incidence and length of hospital stay according to frailty category

	FRAIL					mFI-5						GFI			
	Frail *n* = 37	Non-frail *n* = 166	*p* value				Frail *n* = 78	Non-frail *n* = 125	*p* value				Frail *n* = 66	Non-frail *n* = 137	*p* value
Time to first flatus (h, mean ± SD)	58 ± 32	55 ± 33	0.523				55 ± 35	55 ± 31	0.984				58 ± 33	54 ± 32	0.370
Time to first defecation (h, mean ± SD)	92 ± 51	87 ± 39	0.565				93 ± 49	85 ± 36	0.141				92 ± 48	86 ± 38	0.341
Time to first soft diet intake (h, mean ± SD)	87 ± 49	66 ± 46	0.012*				85 ± 48	61 ± 45	< 0.001*				88 ± 46	61 ± 46	< 0.001*
PPOI (n, %)	22 (59.5)	73 (44.0)	0.088				52 (66.7)	49 (34.4)	< 0.001*				44 (66.7)	51 (37.2)	< 0.001*
Length of hospital stay (d, mean ± SD)	18 ± 9	13 ± 5	< 0.001*				17 ± 8	13 ± 5	< 0.001*				17 ± 7	13 ± 5	< 0.001*

*PPOI* prolonged postoperative ileus, *mFI-5* five-point modified frailty index, *GFI* Groningen Frailty Indicator

*p < 0.05, significant differences between the two groups

### Comparison of the three frailty scales for PPOI prediction

Table 3 shows that COPD, FRAIL ≥ 3, mFI-5 ≥ 2, GFI ≥ 4, surgical type, longer duration of surgery, higher volume of intraoperative fluid infusion, and higher volume of blood loss might be potential predictors of PPOI (*P* < 0.20). The multiple regression analysis indicated that older adults classified as frail by mFI-5 [Odds ratio (OR) 3.230, 95% confidence interval (CI) 1.572–6.638, *P* = 0.001] and GFI (OR 2.627, 95% CI 1.307–5.281, *P* = 0.007) frailty instruments were at a higher risk for PPOI after adjusting the above confounders (Table 4).

**Table 3:** Potential Risk Factors for PPOI by Univariate Analysis

Variables	PPOI group *n* = 95	Non-PPOI group *n* = 108	*p* value
Age (years, mean ± SD)	72.9 ± 5.7	72.3 ± 5.8	0.497
Male (n, %)	52 (54.7)	59 (54.6)	0.988
BMI (Kg/m^2^, mean ± SD)	22.6 ± 3.6	22.5 ± 3.0	0.839
Smoking (n, %)	34 (35.8)	36 (33.3)	0.713
Alcohol consumption (n, %)	46 (48.4)	54 (50.0)	0.822
Previous abdominal surgery (n, %)	20 (21.1)	17 (15.7)	0.328
Hypertension (n, %)	45 (47.4)	45 (41.7)	0.415
Diabetes (n, %)	14 (14.7)	17 (15.7)	0.843
Coronary heart disease (n, %)	7 (7.4)	10 (9.3)	0.627
COPD (n, %)	43 (45.3)	30 (27.8)	0.010*
Cerebral vascular disease (n, %)	7 (7.4)	8 (7.4)	0.992
ASA physical status (n, %)			0.237
I	11 (11.6)	21 (19.4)	
II	64 (67.4)	72 (66.7)	
III	19 (20.0)	13 (12.0)	
IV	1 (1.1)	2 (1.9)	
FRAIL ≥ 3 (n, %)	22 (23.2)	15 (13.9)	0.088
mFI-5 ≥ 2 (n, %)	52 (54.7)	26 (24.1)	< 0.001*
GFI ≥ 4 (n, %)	44 (46.3)	22 (20.4)	< 0.001*
Surgical type (n, %)			0.135
Gastric	25 (26.3)	16 (14.8)	
Small bowel	4 (4.2)	2 (1.9)	
Colonic	46 (48.4)	64 (59.3)	
Rectal	20 (21.1)	26 (24.1)	
Duration of surgery (min, mean ± SD)	228 ± 83	192 ± 75	0.002*
Intraoperative fluid infusion (mL/Kg·h, mean ± SD)	7.0 ± 2.6	7.5 ± 2.4	0.107
Intraoperative packed RBC infusion [mL, median (range)]	0 (0, 800)	0 (0, 600)	0.277
Urine output (mL/Kg·h, mean ± SD)	1.8 ± 2.4	1.9 ± 1.8	0.876
Blood loss [mL, median (range)]	50 (10, 600)	50 (5, 400)	< 0.001*

*ASA* American Society of Anesthesiologists, *BMI* body mass index, *COPD* chronic obstructive pulmonary disease, *FFP* fresh frozen plasma, *GFI* Groningen Frailty Indicator, *mFI-5* five-point modified frailty index, *PPOI* prolonged postoperative ileus, *RBC* red blood cell, *SD* standard deviations

*p < 0.05, significant differences between the two groups

**Table 4:** Risk factors for PPOI in multivariate logistic analysis

								mFI-5 ≥ 2				GFI ≥ 4		
		OR		95%CI		*p* value		OR	95%CI	*p* value		OR	95%CI	*p* value
COPD	No	1						1				1		
	Yes	2.081		1.105–3.918		0.023		1.236	0.595–2.565	0.570		1.551	0.786–3.058	0.206
FRAIL ≥ 3	No	1						NA	NA	NA		NA	NA	NA
	Yes	1.409		0.625–3.174		0.408								
mFI-5 ≥ 2	No	NA		NA		NA		1				NA	NA	NA
	Yes							3.230	1.572–6.638	0.001				
GFI ≥ 4	No	NA		NA		NA		NA	NA	NA		1		
	Yes											2.627	1.307–5.281	0.007
Surgical type	Gastric	1						1				1		
	Small bowel	3.301		0.455–23.932		0.237		2.337	0.300-18.213	0.418		3.221	0.435–23.851	0.252
	Colonic	0.612		0.276–1.356		0.226		0.539	0.238–1.224	0.140		0.648	0.289–1.454	0.293
	Rectal	0.787		0.305–2.027		0.620		0.691	0.261–1.830	0.457		0.772	0.295–2.018	0.597
Duration of surgery	per min increase	1.002		0.997–1.007		0.370		1.001	0.996–1.006	0.711		1.002	0.997–1.007	0.511
Intraoperative fluid infusion	per mL increase	0.935		0.815–1.072		0.334		0.921	0.803–1.056	0.236		0.924	0.806–1.060	0.259
Blood loss	per mL increase	1.005		1.000-1.009		0.054		1.005	1.000-1.009	0.051		1.005	1.000-1.009	0.052

*ASA* American Society of Anesthesiologists, *CI* confidence interval, *COPD* chronic obstructive pulmonary disease, *GFI* Groningen Frailty Indicator, *mFI-5* five-point modified frailty index, *OR* odds ratio, *PPOI* prolonged postoperative ileus

**Fig. 2:**
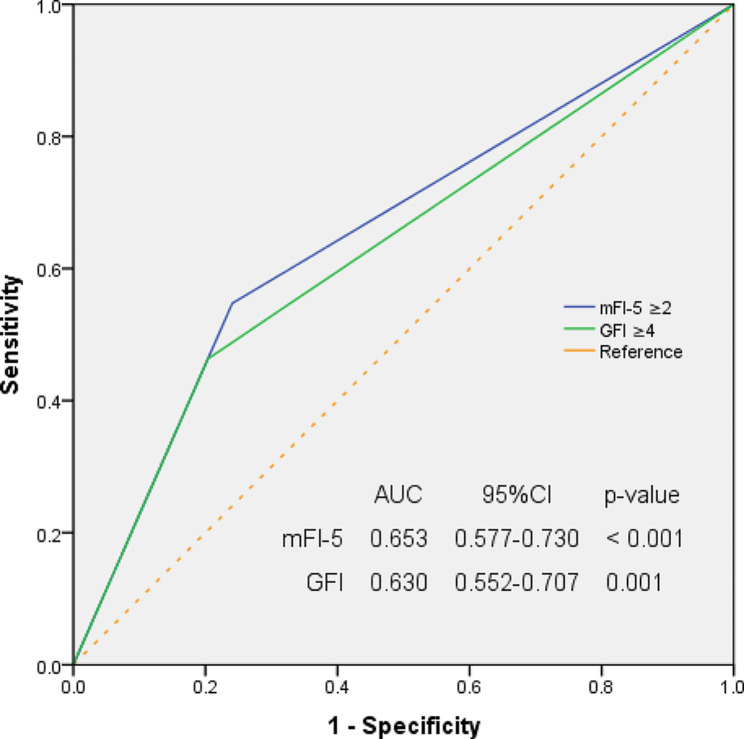
Receiver operating characteristic (ROC) curves of mFI-5 and GFI frailty models for predicting PPOI. Frailty evaluated by both mFI-5 (AUC 0.653, 95% CI 0.577–0.730) and GFI (AUC 0.630, 95% CI 0.552–0.707) scales had insufficient accuracy for the prediction of PPOI

As Fig. 2 presents, the ROC curve analysis indicated that frailty measured by mFI-5 (AUC 0.653, 95% CI 0.577–0.730) or GFI (AUC 0.630, 95% CI 0.552–0.707) scales had insufficient accuracy for the prediction of PPOI.

## Discussion

In this study, we found that frailty diagnosed by FRAIL, mFI-5 or GFI instruments was related with delayed recovery of bowel function. Moreover, an increased risk of PPOI following major abdominal surgery in the elderly patients with frailty identified by mFI-5 and GFI. However, both mFI-5 and GFI cannot accurately predict the occurrence of PPOI following surgery in this population.

Due to differences in patient population and the variety of frailty measures used, widely varying frailty prevalence data has also been reported among surgical patients [36–38]. However, the frailty prevalence in our cohort is consistent with most of the previous studies [39–41]. By using the mFI-5 scale, 38.4% of our cohort was diagnosed with frailty, whereas 18.2% would have been frail by adopting FRAIL scale. We speculated that the omission of social or psychological domains, as measured in the mFI-5 or GFI, might lead to the FRAIL scale’s lower detection rates of frailty.

More and more attention has been paid on preoperative frailty because it is closely related with adverse patient outcome. The present study suggests that simple frailty measures (mFI-5 and GFI) are useful to identify older patients with a higher risk of PPOI after major abdominal surgery, which could benefit institutions with limited access to geriatricians. However, current approaches to identify high-risk patients preoperatively do not usually include frailty assessment [42]. Most currently available surgical risk measures, such as ASA physical status classification, are based on preoperative comorbidities. Complementary indices have been developed to evaluate pulmonary and cardiac risk, but those approaches do not include important aspects including functional status and strength which frailty scales capture [43, 44]. Nor do they assess the patient’s overall functional vulnerability to stress, which frailty scales assess more comprehensively [45, 46]. Therefore, our results add to the evidence documenting the necessity of preoperative frailty evaluation in elderly patients undergoing surgery.

Although there has been no consensus on how to determine and categorize frailty, frailty is increasingly being applied in surgical risk evaluation strategies [47]. The predictive ability for adverse outcomes is perhaps one of the most important characteristics of any risk stratification variable or system. In this study, we found that frailty according to mFI-5 scale was associated with the 3.230 times of odds of PPOI occurrence. Gong et al. demonstrated that higher mFI score was associated with higher risk of delayed recovery of bowel function in patients who undergoing colorectal surgery [48]. A meta-analysis demonstrated that frailty was the strongest preoperative risk factor of postoperative complications. Other factors like age, and ASA grades were not predictive across studies [49]. These findings are consistent with the multivariable analysis of this study which found that frailty was a significant risk factor of PPOI. Considering the high occurrence of preoperative frailty in older surgical patients, and the relevant poor outcome burden, routine evaluation and determination of older patients with frailty prior to the surgery should be proposed.

Frailty is currently recognized as a set of modifiable risk factors. Several studies in older surgical adults have proved that frail patients may benefit from a comprehensive evaluation of geriatrics and specialized processes of care, which highlights the necessity of coordination of transitions of geriatric care and follow-up in this population [50–53]. Individuals identified as frail preoperatively can be targeted for specialized interventions to improve postoperative outcomes.

In the present study, we found that frailty evaluated by FRAIL scale was not an independently risk factor of PPOI. Furthermore, both mFI-5 and GFI score did not perform accurate enough to predict PPOI by ROC analysis in older patients undergoing major abdominal surgery. Our results demonstrate that those three frailty scales may not be ideal screening tools to identify older individuals who have a high risk of PPOI. As a result, new geriatric-specific frailty assessment instruments should be developed to sufficiently predict patient-centered outcomes including delayed recovery of bowel function after operation. On the other hand, our findings also highlight the requirement for further studies that include comparisons between variable frailty measurements (e.g. G8 questionnaire [54], Frailty Index [55], Tilburg Frailty Indicator [56], which are widely used and validated in oncological patients or surgical patients [57–60]), and for the research to consider effect sizes, prediction accuracy, and pragmatic considerations like feasibility, importance and efficiency.

This study has several major limitations. Firstly, our results were generated from a single-center study, which may limit its external generality. Secondly, only three of the commonly used frailty instruments were evaluated. Nonetheless, FRAIL, mFI-5 and GFI are considered as the most-studied and most-robust measures based on the current evidence as they have been proved to have ability in predicting postoperative morbidity in other studies. Thirdly, several types of abdominal surgical procedure were included in this study. This of course possibly results in a heterogeneity of acquired data as the recovery of postoperative bowel movement functions are influenced by surgical type. Lastly, this study was limited to patients undergoing laparoscopic surgery, which limits the generalizability of the results to patients receiving open abdominal procedure.

## Conclusion

In conclusion, we found that 46.8% of older patients experienced PPOI after elective major abdominal surgery; patients who are diagnosed as frail on the mFI-5 and GFI scales are at an increased risk of PPOI. Although frailty might represent a key aspect of preoperative assessment of elderly individual, we demonstrated that neither mFI-5 nor GFI were accurate at identifying elderly surgical patients who will develop PPOI. Future research is needed to determine feasible and accurate preoperative frailty screening scales for delayed recovery of bowel function in this population.

## Data Availability

The data supporting the findings of this study are available upon contacting the corresponding author.

## Abbreviations

ASA: American society of anesthesiologists
AUC: Area under curve
BMI: Body mass index
CI: Confidence interval
COPD: Chronic obstructive pulmonary disease
FFP: Fresh frozen plasma
GFI: Groningen frailty indicator
mFI-5: Five-point modified frailty index
OR: Odds ratio
PACU: Post-anesthesia care unit
PCA: Patient-controlled analgesia
POD: Postoperative day
POI: Postoperative ileus
PPOI: Prolonged postoperative ileus
RBC: Red blood cell
ROC: Receiver operating characteristic
SD: Standard deviation
PPOI: prolonged postoperative ileus
mFI-5: five-point modified frailty index
GFI: Groningen frailty indicator
AUC: area under curve
CI: confidence interval
